# Hybridization, Maternal Inheritance, and Evolutionary Time of Divergence of Endangered Species of *Hypancistrus* (Siluriformes: Loricariidae) in the Xingu River

**DOI:** 10.1002/ece3.73624

**Published:** 2026-05-14

**Authors:** Franciele F. Kerniske, Bruno F. Melo, Leandro M. Sousa, Tiago M. Degrandi, Roberto F. Artoni

**Affiliations:** ^1^ Programa de Pós‐Graduação em Genética Evolutiva e Biologia Molecular Universidade Federal de São Carlos (UFSCar) São Carlos SP Brazil; ^2^ Division of Vertebrate Zoology (Ichthyology) American Museum of Natural History New York New York USA; ^3^ Laboratório de Ictiologia de Altamira Universidade Federal do Pará (UFPA) Altamira PA Brazil; ^4^ Programa de Pós‐Graduação em Biologia Evolutiva Universidade Estadual de Ponta Grossa (UEPG) Ponta Grossa PR Brazil

**Keywords:** Amazon fishes, Belo Monte hydropower dam, conservation, genome size, hybrids, mitogenome

## Abstract

Catfishes of the genus *Hypancistrus* are among the most emblematic endemic species of the Xingu River rapids, a biodiversity hotspot of the Amazon Basin. In the unique Volta Grande region, the distribution of *Hypancistrus zebra*, *Hypancistrus seideli*, and *Hypancistrus yudja* overlap, creating a potential zone of biological interaction, and previous morphological analyses suggest the occurrence of hybridization between *H. seideli* and *H. yudja*. We investigated maternal inheritance and phylogenetic relationships among the three species and hybrids using complete mitochondrial genomes and nuclear genome size estimates. Phylogenetic analyses revealed that hybrids carried maternal lineages exclusively from *H. zebra* and *H. seideli*, with no evidence of contribution from *H. yudja*. Nuclear genome size analyses support this pattern, with hybrids exhibiting intermediate values consistent with additive inheritance. Divergence time estimates indicated a very recent evolutionary radiation (< 0.5 Ma), explaining the persistence of reproductive compatibility among the species. These findings raise conservation concerns, particularly for the zebra pleco *H. zebra* , a critically endangered species at risk of genetic introgression. Our study provides the first complete mitochondrial genome data for these species and emphasizes the importance of integrating morphological and genomic approaches to understand hybridization dynamics and guide conservation strategies in the Xingu River.

## Graphical abstract

We uncover recent hybridization dynamics among three endemic Hypancistrus species from the Xingu River, including the critically endangered *H. zebra* . Mitogenomic analyses reveal unidirectional maternal inheritance and recent evolutionary radiation (< 0.5 Ma), explaining their retained reproductive compatibility. These findings highlight the evolutionary and conservation significance of these Amazonian lineages.

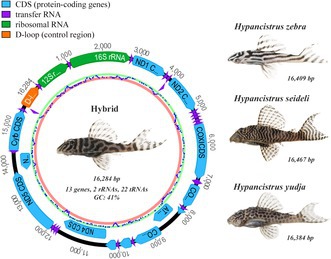

## 1 Introduction

The Xingu River in the eastern Amazon basin is well‐known for its extraordinary biodiversity and high levels of ichthyological endemism (Sabaj et al. 2024). The stretch of the Volta Grande do Xingu, a mosaic of rapids, rocky outcrops, and clear waters, hosts a unique and highly adapted ichthyofauna, including species of the genus *Hypancistrus* (Loricariidae) (Santos 2019). Among these, *Hypancistrus zebra* (Isbrücker and Nijssen 1991), *Hypancistrus seideli* (Sousa et al. 2025), and *Hypancistrus yudja* (Sousa et al. 2025) are notable for their ecological significance and commercial value in the ornamental fish trade (Gonçalves 2020; Sousa et al. 2021, 2025). The complexity of this hydrologic system presents significant challenges for understanding evolutionary processes and conservation efforts for these endemic species (Zuanon 1999; Goulding et al. 2003).

A recent study based on geometric morphometrics indicates the lower portion of the Volta Grande do Xingu as a potential hybridization zone involving *H. seideli* and *H. yudja* (Kerniske et al. 2025), whose current dynamics may be influenced by recent environmental events, such as the construction of the Belo Monte Hydroelectric Dam and extreme droughts that alter the hydrological regime and habitat connectivity (Fitzgerald et al. 2018; Jiang et al. 2018). This contemporary scenario is rooted in a deep evolutionary context, including Neogene and Quaternary hydrological events shaping the Amazon Basin's current configuration, promoting cycles of river course changes and altering basin connectivity (Albert and Reis 2011; Hoorn et al. 2017; Albert et al. 2018). Cycles of isolation and subsequent reconnection of aquatic populations resulted in conditions conducive to natural hybridization and diversification processes among species (Albert and Reis 2011; Hoorn et al. 2017). Similar patterns have been observed in *Rhamdia*, a group of Siluriformes that underwent diversification and colonization of cave environments in distinct hydrographic systems of Mexico and Central America during the Pleistocene (~2 Ma) (Arroyave et al. 2025). These findings reinforce that processes such as drainage fragmentation and the formation of ecological refugia may have promoted rapid and independent radiation across distinct Neotropical lineages (Arroyave et al. 2025).

Historical evidence from the ornamental trade reinforces that morphotypes of *H. zebra* and morphologically unusual variants, known as “mimics” have been collected and exported from the Xingu to the international market since the late 1980s (Seidel and Evers 2005; Seidel 2008; Camargo et al. 2013; Haagensen 2014; Evers et al. 2019). Documented in specialized catalogs, these *Hypancistrus*, identified by L‐number codes, often did not correspond directly to formally described species (Stawikowski 1990; Glaser and Glaser 1995; Seidel and Evers 2005). In this context, the ornamental fishery emerges as a source of anthropogenic impact associated with hybridization, favoring both the selective removal of rare morphotypes and the manipulation of natural species distributions, including the potential artificial introduction of *H. seideli* upstream of geographic barriers in the Volta Grande do Xingu. This perspective contributes to a broader understanding of the role of natural hybridization in generating genetic diversity and shaping the evolutionary dynamics of Xingu fish populations, and highlights the need for modern genomic analyses to elucidate historical and contemporary patterns of species diversification.

Hybridization is a complex phenomenon that acts as a source of genetic variation and adaptation but may also compromise the genetic integrity of parental species, potentially leading to the loss of unique evolutionary lineages and the decline of already vulnerable populations (Mallet 2007). In the *Hypancistrus* system of the Xingu River, the hybridization zone is localized within the Volta Grande region, with limited spatial overlap among species (Kerniske et al. 2025). This contact zone represents an exception to the predominantly allopatric distribution pattern observed (Sousa et al. 2025), and may reflect recent geomorphological events that facilitated secondary contact between historically‐isolated lineages. In particular, *H. yudja* appears to be more susceptible to genetic impacts due to its highly specialized and restricted distribution compared to *H. seideli* or *H. zebra* (Kerniske et al. 2025; Sousa et al. 2025). Therefore, understanding the frequency, directionality of crosses, and the contribution of each parental species is essential to clarify the dynamics of hybrid populations.

Directional crossing, often asymmetric, has been widely documented for teleosts, with significantly greater gene flow from one species to another (Barton and Hewitt 1989; Wirtz 1999). This pattern may reflect prezygotic barriers, such as differences in mating behavior and female mate choice, or postzygotic barriers, such as reduced viability or fertility of hybrids in only one direction of the cross (Arnold 1997; Coyne and Orr 2004). Examples of unidirectional hybridization, such as in cichlids from Lake Malawi where females preferentially select males with specific coloration patterns (Seehausen et al. 1997), and bidirectional cases, as observed in sympatric *Plectropomus* species with fertile hybrids over multiple generations (Harrison et al. 2017), illustrate the diversity of these patterns. Within Siluriformes, hybridization between *Pseudoplatystoma corruscans* and *P. reticulatum* (Pimelodidae) has been reported in the Paraná‐Paraguay basin, although largely associated with aquaculture escapes (Prado et al. 2011). In contrast, natural in situ hybridization driven by environmental change within Loricariidae remains undocumented, making the present study a potentially pioneering case within the family. In this context, analyses using mitochondrial DNA have been fundamental to elucidate the dynamics of such hybridization events, enabling the identification of gene flow direction and maternal inheritance (Moritz et al. 1987; Galtier et al. 2009; Avise 2000; Toews and Brelsford 2012).

In this study, we analyzed mitochondrial genomes (mitogenome) of the three *Hypancistrus* and related loricariids to determine the maternal lineage of species and hybrids and infer phylogenetic relationships among species. Additionally, we estimated the average nuclear genome size, an important metric for comparative genomic studies, evolutionary time analyses, and the planning of broader genomic approaches. These data provide novel resources for understanding evolutionary dynamics and for developing effective conservation strategies for these species in their natural environments.

## 2 Material and Methods

### 2.1 Sampling and Species Studied

This study analyzed three endemic *Hypancistrus* species collected in the Volta Grande region of the Xingu (Altamira, Brazil): *H. zebra* (*N* = 3), *H. yudja* (*N* = 3), *H. seideli* (*N* = 5), in addition to individuals previously identified as putative hybrids (*N* = 5), according to Kerniske et al. (2025). The specimens were maintained in tanks of the Xingu Aquaculture Laboratory (LAQUAX) at the Universidade Federal do Pará (UFPA) under controlled conditions. It is important to clarify that the five hybrids analyzed here are the same ones identified by Kerniske et al. (2025) using morphological approaches, whereas the parental species represent independent samples reflecting the mitochondrial genetic diversity reported for each lineage. The reduced sample sizes reflect the critically endangered or restricted status of these species: collection is tightly regulated under SISBIO permit No. 79124‐1, and recent environmental monitoring and diving surveys conducted by the LAQUAX team at UFPA (Sousa et al. 2025) detected a significant decline in the abundance of *H. yudja* relative to the other *Hypancistrus* species, consistent with an estimated population decline of over 80% and a possible Critically Endangered (CR) classification. This rarity directly constrains the number of specimens available for destructive sampling. From an analytical standpoint, mitochondrial genome analyses are particularly informative even with small sample sizes when the objective is to determine maternal lineage assignment, because haplotype diversity in recently diverged taxa tends to be low and phylogenetically structured (Toews and Brelsford 2012). Phylogeographic studies of other Xingu Loricariidae confirm limited mitochondrial diversity within species at fine spatial scales (Magalhães et al. 2021), supporting the adequacy of our sampling for inferring maternal inheritance patterns.

The research was conducted following the guidelines of the Conselho Nacional de Controle da Experimentação Animal (CONCEA). It was authorized by the Comissão de Ética no Uso de Animais da Universidade Federal do Pará (protocol CEUA No. 6895300622) and the collection license of SISBIO/MMA No. 79124‐1.

### 2.2 Genomic DNA Extraction and Next‐Generation Sequencing (NGS)

Genomic DNA (gDNA) was extracted from dorsal fin tissues using the phenol‐chloroform protocol (Sambrook and Russell 2006). The integrity of the gDNA was verified by 1% agarose gel electrophoresis and concentration quantified by Nanovue spectrophotometer. Following extraction, genomic DNA was fragmented, and paired‐end libraries were constructed according to the standard BGI Whole Genome Resequencing (WGRS) protocol, which involves linear isothermal rolling‐circle replication to form DNA Nanoballs (DNBs). The 16 *Hypancistrus* libraries were then sequenced on the DNBSEQ (BGI) platform, generating approximately 2 Gb of raw data in 150‐bp paired‐end reads per sample.

### 2.3 Mitochondrial Genome Assembly

The quality of the raw data was assessed using FastQC and pre‐processed with Trimmomatic (Bolger et al. 2014) to remove sequencing adapters and filter reads by quality control. Mitochondrial genomes were assembled using GetOrganelle v. 1.7.6.1 (Jin et al. 2020), following established protocols and using reference mitogenomes from representatives of the family Loricariidae available in GenBank. The reference sequences used were: *Pterygoplichthys disjunctivus* (NC_015747), *Hypostomus plecostomus* (NC_025584), *Hypoptopoma incognitum* (NC_028072), *Hypostomus francisci* (NC_045188), *Sturisomatichthys panamensis* (NC_045877), *Ancistrus temminckii* (NC_051963), *Hypostomus ancistroides* (NC_052710), and *Pterygoplichthys pardalis* (NC_060468). Protein‐coding genes (PCGs), ribosomal RNA genes (rRNAs), transfer RNA genes (tRNAs), and the control region (D‐loop) were annotated using MitoFish v3.98 and MitoAnnotator (Iwasaki et al. 2013; Zhu et al. 2023) provided by MitoFish v.3.98 (Zhu et al. 2023). Final assemblies were manually checked and edited in Geneious v7.1.3 (Kearse et al. 2012).

### 2.4 Phylogenetic Analyses

We used the 13 protein‐coding genes (PCGs) annotated and aligned across all individuals. Sequences for each gene were aligned individually using the MUSCLE algorithm (Edgar 2004) implemented in the online service of the European Molecular Biology Laboratory—European Bioinformatics Institute (EMBL‐EBI) at https://www.ebi.ac.uk/jdispatcher/msa/muscle. The concatenated alignments generated a matrix of 11,416 bp for the 13 protein‐coding genes (PCGs).

Maximum likelihood phylogenetic trees were then reconstructed using the IQ‐TREE software (version 1.6.12) (Nguyen et al. 2015). A partitioned model based on the 13 mitochondrial protein‐coding genes (PCGs) was applied, totaling 11,416 aligned sites. ModelFinder (Kalyaanamoorthy et al. 2017) was used to select the best nucleotide substitution model for each partition, based on the Bayesian Information Criterion (BIC). Branch support was evaluated using ultrafast bootstrap (UFBoot) with 1000 pseudoreplicates (Hoang et al. 2018) and the Shimodaira‐Hasegawa approximate likelihood ratio test (SH‐aLRT) with 1000 replicates. The trees were rooted using *A. temminckii* (Valenciennes 1840) and *H. ancistroides* (von Ihering 1911) as outgroups. These taxa were selected because they represent well‐characterized lineages within the subfamily Hypostominae that are phylogenetically external to the *Hypancistrus* clade, according to the family‐level phylogeny of Roxo et al. (2019), providing an appropriate root for divergence time estimation within the genus *Hypancistrus*.

### 2.5 Divergence Time Estimation

Divergence time estimation for *Hypancistrus* species were estimated using a Bayesian molecular clock approach in BEAST v2.5 (Bouckaert et al. 2019). A sequence alignment matrix with 20 terminals was built containing 10,274 bp from the 13 protein‐coding genes. We included mitogenomes of the three species of *Hypancistrus* and available mitogenomes of related genera of the subfamily Hypostominae such as *Ancistrus*, *Hypostomus*, *Pterygoplichthys* and other loricariids such as *Hypoptopoma*, *Otocinclus*, and *Sturisomatichthys* as root, following the loricariid phylogeny based on genomic data (Roxo et al. 2019). The analysis employed a relaxed lognormal molecular clock (mean clock.rate = 1.0), the birth‐death tree model (death rate = 0.5; birth rate = 1.0), and the GTR + G + I site model (gamma category count = 4; invariant sites = 0.5). Due to incomplete fossil record of genera with available mitogenomes, we used two secondary calibrations following the time‐calibrated phylogeny of Loricariidae (Roxo et al. 2019). The first calibration was applied to the node corresponding to the divergence between the main clades of Loricariidae (Hypoptopomatinae and Hypostominae), with mean age in the Early Miocene (mean = 17.0; sigma = 3.0; 95%–5% quantiles: 21.9–12.1). This node includes *Hypoptopoma*, *Otocinclus* and *Sturisomatichthys* as representatives of Hypoptopomatinae. The second calibration included a root constraint that included all loricariids of the analysis dated to Eocene–Oligocene boundary (mean = 42.5; sigma = 8.0; 95%–5% quantiles: 55.7–29.3) following estimations of Roxo et al. (2019). Markov Chain Monte Carlo (MCMC) chains were run for 100 million generations, sampling every 10,000 generations. Convergence was assessed in Tracer v1.7.2 (Rambaut et al. 2018), ensuring ESS values > 200 for all parameters. The maximum clade credibility (MCC) tree was generated from the last 9001 trees using TreeAnnotator, discarding 10% as burn‐in (1000 trees), and visualized with iTOL v6 (Letunic and Bork 2024). Alignments and input files are available in the Dryad Digital Repository (https://doi.org/10.5061/dryad.7pvmcvf6p).

### 2.6 Genome Size Estimation

Nuclear genome size estimates were performed using a Sysmex PA Ploidy Analyzer flow cytometer. Fin tissue samples (*H. seideli*, *H. yudja*, *H. zebra* , and hybrids), preserved in DMEM, were mechanically homogenized and treated with lysis solution (0.8% Triton X‐100). The cell suspension was filtered, stained with DAPI CyStain UV Ploid (Xavier et al. 2017), and analyzed using *Astyanax mexicanus* (De Filippi 1853) (Characiformes: Acestrorhamphidae) as an internal standard. The haploid genome size of the internal standard, *A. mexicanus* (1.4 Gb), was used for calibration, based on the genome assembly ASM2337597v1 (GCF_023375975.1) (NCBI 2025). The genome size (Gb) was calculated using the formula:

$$\text{Sample size}=\left(\text{DAPI}\;{\text{median}}^{\text{sample}}/\text{DAPI}\;{\text{median}}^{\text{standard}}\right)\times 1.4$$

## 3 Results

### 3.1 Mitochondrial Genome Characterization

**FIGURE 1:**
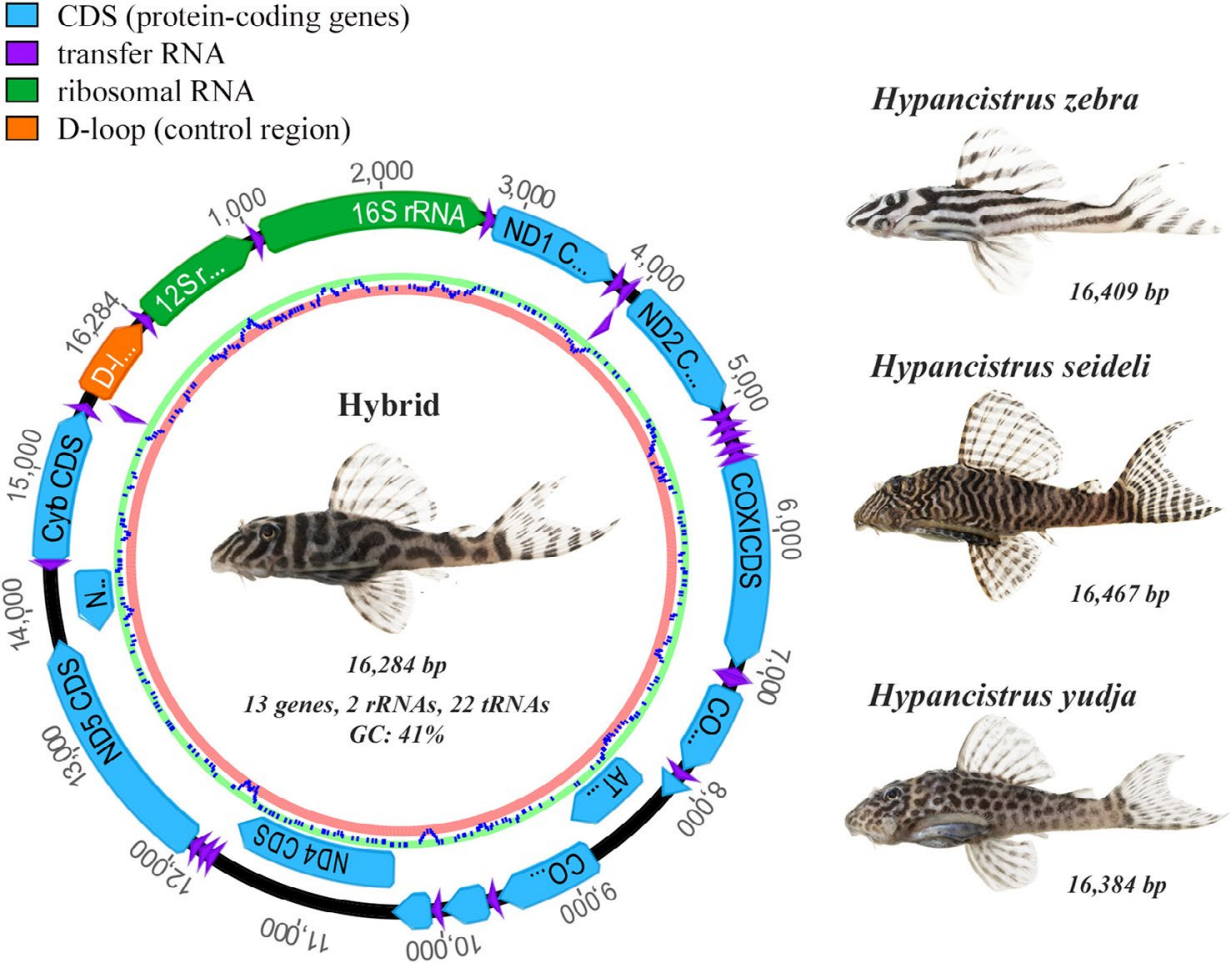
Circular organization of the complete mitochondrial genome of the hybrid of *Hypancistrus*, and photographs of the three parental species—*H. zebra, H. seideli*, and *H. yudja*—with their respective mitochondrial genome sizes. Photos by Leandro M. Sousa.

The genomic organization of the mitochondrial DNA followed the typical vertebrate pattern for all individuals, including 13 protein‐coding genes, 2 rRNA genes, and 22 tRNA genes, as well as the control region (D‐loop). The total mitochondrial genome size ranged from 16,384 bp in *H. yudja* to 16,467 bp in *H. seideli*, with a GC content of approximately 41%. These data represent the first detailed characterization of the mitochondrial genome for these three *Hypancistrus* species (Table 1, Figure 1).

**TABLE 1:** Annotation of mitochondrial genome of *Hypancistrus* species analyzed in this study, including gene names, strand orientation, sequence positions (start and end), gene size (bp), and start/stop codons (for protein‐coding genes).

Gene/element	Strand	Position start/end				Size (bp)				Codon start/stop			
		*H. zebra*	*H. seideli*	*H. yudja*	Hybrid	*H. zebra*	*H. seideli*	*H. yudja*	Hybrid	*H. zebra*	*H. seideli*	*H. yudja*	Hybrid
tRNA‐Phe	H	1–68	1–68	1–68	1–68	68	68	68	68				
12S‐rRNA	H	69–1016	69–1016	69–1016	69–1016	948	948	948	948				
tRNA‐Val	H	1017–1088	1017–1088	1017–1088	1017–1088	72	72	72	72				
16S‐rRNA	H	1089–2768	1089–2764	1089–2766	1089–2768	1680	1676	1678	1680				
tRNA‐Leu	H	2769–2843	2765–2839	2767–2841	2769–2843	75	75	75	75				
ND1	H	2844–3818	2840–3814	2842–3816	2844–3818	975	975	975	975	ATG/TAA	ATG/TAA	ATG/TAA	ATG/TAA
tRNA‐Ile	H	3823–3894	3819–3890	3821–3892	3823–3894	72	72	72	72				
tRNA‐Gln	L	3895–3965	3891–3961	3893–3963	3895–3965	71	71	71	71				
tRNA‐Met	H	3965–4034	3961–4030	3963–4032	3965–4034	70	70	70	70				
ND2	H	4035–5079	4031–5075	4033–5077	4035–5079	1045	1045	1045	1045	ATG/T‐‐	ATG/T‐‐	ATG/T‐‐	ATG/T‐‐
tRNA‐Trp	H	5080–5150	5076–5146	5078–5148	5080–5150	71	71	71	71				
tRNA‐Ala	L	5153–5221	5149–5217	5151–5219	5153–5221	69	69	69	69				
tRNA‐Asn	L	5223–5295	5219–5291	5221–5293	5223–5295	73	73	73	73				
tRNA‐Cys	L	5328–5394	5324–5389	5326–5392	5328–5394	67	66	67	67				
tRNA‐Tyr	L	5395–5464	5390–5459	5393–5462	5395–5464	70	70	70	70				
COXI	H	5466–7016	5461–7011	5464–7014	5466–7016	1551	1551	1551	1551	GTG/TAA	GTG/TAA	GTG/TAA	GTG/TAA
tRNA‐Ser	L	7017–7087	7012–7082	7015–7085	7017–7087	71	71	71	71				
tRNA‐Asp	H	7092–7164	7087–7159	7090–7162	7092–7164	73	73	73	73				
COXII	H	7179–7869	7174–7864	7177–7867	7179–7869	691	691	691	691	ATG/T‐‐	ATG/T‐‐	ATG/T‐‐	ATG/T‐‐
tRNA‐Lys	H	7870–7943	7865–7938	7868–7941	7870–7943	74	74	74	74				
ATPase8	H	7945–8112	7940–8107	7943–8110	7945–8112	168	168	168	168	ATG/TAA	ATG/TAA	ATG/TAA	ATG/TAA
ATPase6	H	8103–8785	8098–8780	8101–8783	8103–8785	683	683	683	683	ATG/TA—	ATG/TA—	ATG/TA—	ATG/TA—
COXIII	H	8786–9569	8781–9564	8784–9567	8786–9569	784	784	784	784	ATG/T‐‐	ATG/T‐‐	ATG/T‐‐	ATG/T‐‐
tRNA‐Gly	H	9570–9641	9565–9636	9568–9639	9570–9641	72	72	72	72				
ND3	H	9642–9990	9637–9985	9640–9988	9642–9990	349	349	349	349	ATG/T‐‐	ATG/T‐‐	ATG/T‐‐	ATG/T‐‐
tRNA‐Arg	H	9991–10,060	9986–10,055	9989–10,058	9991–10,060	70	70	70	70				
ND4L	H	10,061–10,357	10,056–10,352	10,059–10,355	10,061–10,357	297	297	297	297	ATG/TAA	ATG/TAA	ATG/TAA	ATG/TAA
ND4	H	10,351–11,731	10,346–11,726	10,349–11,729	10,351–11,731	1381	1381	1381	1381	ATG/T‐‐	ATG/T‐‐	ATG/T‐‐	ATG/T‐‐
tRNA‐His	H	11,732–11,801	11,727–11,796	11,730–11,799	11,732–11,801	70	70	70	70				
tRNA‐Ser	H	11,802–11,870	11,797–11,863	11,800–11,868	11,802–11,870	69	67	69	69				
tRNA‐Leu	H	11,872–11,943	11,865–11,936	11,870–11,941	11,872–11,943	72	72	72	72				
ND5	H	11,944–13,770	11,937–13,763	11,942–13,768	11,944–13,770	1827	1827	1827	1827	ATG/TAA	ATG/TAA	ATG/TAA	ATG/TAA
ND6	L	13,767–14,288	13,760–14,281	13,765–14,286	13,767–14,288	522	522	522	522	ATG/TAG	ATG/TAG	ATG/TAG	ATG/TAG
tRNA‐Glu	L	14,289–14,357	14,282–14,350	14,287–14,355	14,289–14,357	69	69	69	69				
CYTB	H	14,360–15,497	14,353–15,490	14,358–15,495	14,360–15,497	1138	1138	1138	1138	ATG/T‐‐	ATG/T‐‐	ATG/T‐‐	ATG/T‐‐
tRNA‐Thr	H	15,498–15,570	15,491–15,563	15,496–15,568	15,498–15,570	73	73	73	73				
tRNA‐Pro	L	15,569–15,638	15,562–15,631	15,567–15,636	15,569–15,638	70	70	70	70				
D‐loop	H	15,639–16,408	15,632–16,466	15,637–16,383	15,639–16,283	770	835	747	645				

Abbreviations: H, heavy strand; L, light strand.

### 3.2 Maternal Inheritance Revealed by Mitochondrial Phylogenetic Analyses

**FIGURE 2:**
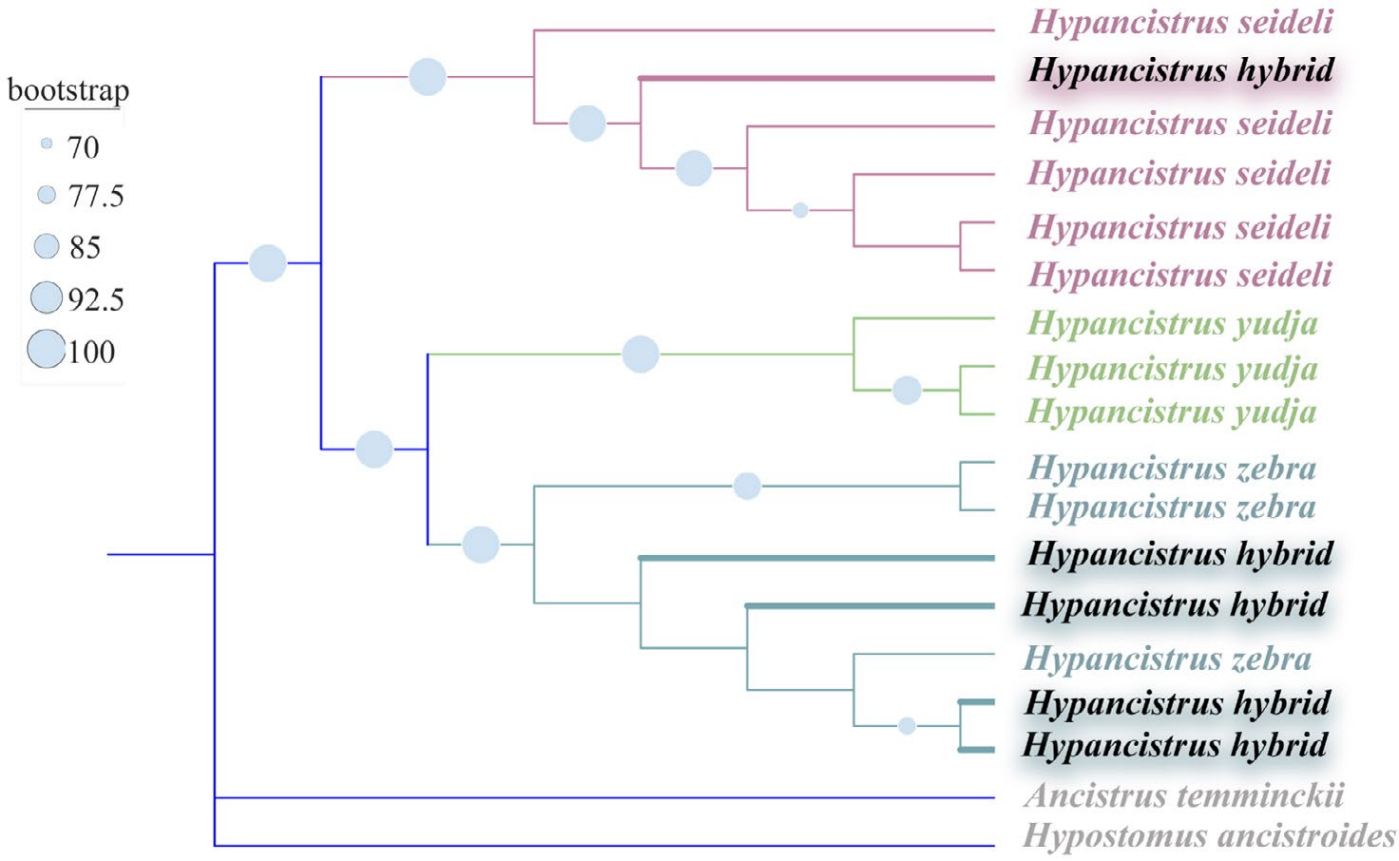
Maximum likelihood phylogeny based on the 13 mitochondrial protein‐coding genes. The analysis shows hybrids grouping with *H. zebra* and *H. seideli*, but not with *H. yudja*. Node support values (SH‐aLRT/UFBoot) are indicated as blue circles of varying sizes alongside nodes.

Phylogenetic analyses based on the 13 protein‐coding genes of the mitogenomes (Figure 2), consistently show that the hybrid *Hypancistrus* individuals exhibit maternal inheritance from *H. zebra* and *H. seideli*, but not from *H. yudja*. Thehybrids H10F, H14F, H16M, and H18M formed a robust clade (100% bootstrap support) with *H. zebra* . Similarly, the hybrid H13F grouped consistently (100% bootstrap support) within the *H. seideli* clade. Regarding the overall phylogenetic inference, the genus *Hypancistrus* received maximum support (SH‐aLRT/UFBoot = 100/100). Within the genus, the clade containing *H. seideli* and related hybrids was also maximally supported (100/100), as was the clade containing *H. yudja* (99.9/100) and the clade containing *H. zebra* and related hybrids (99.9/100). The absence of any hybrid clustering with the *H. yudja* clade in the mitochondrial analysis indicates that *H. yudja* has not been involved in hybridization processes in the Volta Grande region as a maternal lineage.

### 3.3 Divergence Time of *Hypancistrus*

**FIGURE 3:**
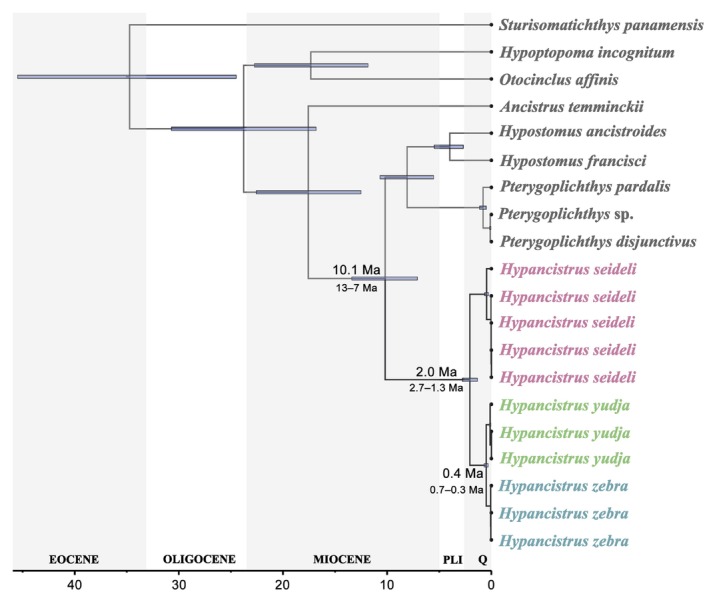
Time‐calibrated phylogeny of *Hypancistrus* and related loricariids based on mitochondrial protein‐coding genes. The tree indicates a Late Miocene split of *Hypancistrus* and the Quaternary diversification (< 2.5 Ma) among *H. zebra, H. seideli*, and *H. yudja*.

The time‐calibrated phylogeny of *Hypancistrus* indicated that the genus diverged from the clade including *Hypostomus* and *Pterygoplichthys* (Hypostominae) during the Tortonian of the Late Miocene at approximately 10.1 million years ago (Ma) (13.4–7.1 Ma 95% highest posterior density HPD) (Figure 3). Diversification within the *Hypancistrus* began with the split of *H. seideli* and the clade with *H. yudja* and *H. zebra* during the Early Pleistocene at around 2.05 Ma (2.78–1.34 Ma 95% HPD), and the speciation process involving *H. yudja* and *H. zebra* is estimated to have occurred during the Middle Pleistocene at *circa* 0.48 Ma (0.68–0.30 Ma 95% HPD).

### 3.4 Genome Size Estimates

Nuclear genome size estimates, obtained via flow cytometry, showed variation among species and hybrids. *H. zebra* had the largest diploid genome (2.8971 Gb), followed by *H. seideli* (2.8170 Gb) and *H. yudja* (2.6741 Gb). The genome of the hybrid individuals was estimated at 2.7427 Gb, an intermediate value between the genomes of the putative parental species.

## 4 Discussion

The results of this study provide a comprehensive understanding of hybridization patterns in *Hypancistrus* species from the Xingu River, with a particular focus on maternal inheritance and conservation implications. Although geometric morphometrics (Kerniske et al. 2025) suggested the occurrence of hybridization between *H. seideli* and *H. yudja*, our analyses based on the mitochondrial genome, conducted on the same hybrid individuals previously examined morphologically, did not detect *H. yudja* maternal lineages in any of the hybrids analyzed. Instead, the mitochondrial haplotypes of the hybrids consistently clustered with *H. zebra* and *H. seideli*.

This discrepancy between morphological and molecular data is a crucial point. Since mitochondrial DNA is exclusively maternally inherited, the absence of *H. yudja* haplotypes indicates that females of this species are not contributing as maternal parents. However, this does not exclude the possibility that *H. yudja* acts as a paternal parent. Hybridization may occur unidirectionally, with one species providing the maternal lineage and the other the paternal lineage (Harrison et al. 2017). In this scenario, *H. zebra* and *H. seideli* females would mate with *H. yudja* males, producing hybrids with intermediate morphology but mitochondrial lineages derived from the maternal species.

It is also important to consider that the limited number of *H. yudja* samples may have been insufficient to capture the full mitochondrial variability of the species. Therefore, we cannot definitively rule out the possibility that *H. yudja* females might act as maternal parents in other, unsampled hybridization events in the wild. However, for the five specific hybrid individuals analyzed in this study, the robust topology of the 13‐gene phylogeny demonstrates that they nest unambiguously within the *H. zebra* and *H. seideli* clades. This consistent topological placement confirms these two species as the maternal lineages for these specific hybrids, effectively ruling out *H. yudja* as their maternal parent.

The recent evolutionary history and phylogenetic relationships among the studied species provide an important context for interpreting hybridization dynamics. Our phylogenetic analysis indicates that *H. seideli* represents the sister group of the clade with *H. yudja* and *H. zebra* that resulted from a more recent diversification event. This topology contrasts with the biogeographic hypothesis proposed by Santos (2019), based on ddRADseq data, according to which *H. yudja* and *H. zebra* would represent the oldest lineage in the Volta Grande, *H. zebra* would have originated by dispersal from the Tapajós River, and *H. seideli* represents a more recently derived lineage resulting from colonization from Guiana Shield drainages, possibly accompanied by introgression. The topology recovered in the present study thus suggests a history marked by recent secondary contact and gene flow in the Volta Grande, providing a coherent evolutionary scenario for the hybridization currently observed.

The paleohydrological history of the Xingu River, marked by cycles of population fragmentation, secondary contact, and reconnection during the Miocene and Pleistocene, created the conditions for both endemism and reproductive contact between closely related lineages (Albert and Reis 2011; Dagosta and de Pinna 2017). This history of isolation followed by secondary contact is directly relevant to interpreting the hybridization dynamics observed here. The recent diversification among these *Hypancistrus* species (with speciation events occurring during the Pleistocene, within the last ~2.0 Ma) suggests that incomplete reproductive isolation is the expected outcome in such a geomorphologically dynamic system. In this context, lineages that experienced vicariance were not isolated long enough to develop complete reproductive barriers before coming into secondary contact.

In addition, the characterization of mitochondrial DNA and estimates of nuclear genome size provide novel data for *Hypancistrus* species from the Xingu River. The observed mitochondrial genome organization was conserved, as is typical in vertebrates (Boore 1999), whereas variations in nuclear genome size suggest differences in genomic complexity among species, with *H. zebra* presenting a genome approximately 8% larger than that of *H. yudja*. Such differences are likely related to the amount and dynamics of repetitive DNA sequences, such as transposable elements and satellite DNA (Gregory 2005; Kidwell 2002). The expansion or contraction of these regions is a major driver of genome evolution (Lynch 2007), and characterizing these elements will be fundamental to understanding the structural bases of such variation. Additionally, the intermediate genome size found in hybrids provides complementary nuclear evidence of genomic admixture between parental species. The hybrid genome size estimate of 2.7427 Gb falls between *H. zebra* (2.8971 Gb) and *H. yudja* (2.6741 Gb), and is similarly intermediate relative to *H. seideli* (2.8170 Gb). This pattern is consistent with additive inheritance of parental genome sizes, a phenomenon expected in F1 hybrids where both parental genomes contribute equally to the nuclear content (Gregory 2005). The morphological data reported in Kerniske et al. (2025) identified these same five individuals as putative hybrids based on intermediate morphology between *H. seideli* and *H. yudja*; the present genome size data provide independent, nuclear‐level corroboration of that morphological inference. Together, the mitochondrial and genome size evidence converge on a consistent hybrid signal, strengthening the overall interpretation. Further analyses with biparental nuclear markers such as SNPs or microsatellites will be essential to determine the generation status and directionality of these hybridization events, and to assess whether the intermediate genome size reflects a first‐generation hybrid condition or the result of multiple backcross events.

Another relevant aspect is the low temporal divergence observed among species. Their recent divergence, occurring within the last 480,000 years, may explain the maintenance of reproductive compatibility, as prezygotic barriers (such as gametic or behavioral isolation) appear insufficient to prevent interbreeding among these lineages. This absence of strong barriers allows *H. zebra* and *H. seideli* individuals to recognize each other and produce viable hybrids even after evolutionary isolation, as corroborated by the occurrence of natural hybrids with intermediate morphology. However, the fertility of these hybrids in natural environments still requires direct investigation. Hybrid viability does not necessarily imply fertility, and subtle genetic incompatibilities may affect gamete production or reproductive success (Maheshwari and Barbash 2011; Lindtke and Buerkle 2015). If hybrids prove fertile and capable of backcrossing with parental species, the conservation implications become more severe: recurrent backcrossing can lead to progressive introgression, gradually eroding the genetic distinctiveness of parental lineages (Mallet 2007; Allendorf et al. 2019). In such scenarios, distinguishing between first‐generation hybrids and backcross individuals becomes crucial for assessing the extent and directionality of gene flow. Although informal observations suggest that these hybrids may be viable and potentially fertile under captive conditions, formal experimental data remain unavailable. Furthermore, while putative hybrids often commercialized as “mimic” variants are documented in the aquarium trade (Seidel and Evers 2005; Evers et al. 2019), hobbyist accounts of their reproduction remain largely anecdotal. Future studies using individuals of known origin, experimental crosses, and biparental markers will be fundamental to clarify the role of these hybrids in the evolutionary dynamics of the species involved. In this context, the patterns of genetic diversification observed in *Hypancistrus* reflect broader evolutionary trends already documented in other Amazonian loricariids (Silva et al. 2016). A parallel example is *Baryancistrus xanthellus*, a rheophilic catfish endemic to the Xingu, in which phylogeographic studies detected strong genetic structuring among populations, shaped by the basin's geomorphological dynamics and marked by cycles of isolation and secondary contact (Magalhães et al. 2021). Furthermore, chromosomal diversification driven by repetitive sequences is a recurrent feature in Amazonian Loricariidae. Comparative studies on genera such as *Ancistrus* (Santos da Silva et al. 2022), *Peckoltia* (Santos da Silva et al. 2021), and *Spatuloricaria* (Almeida et al. 2023) have revealed an intense dynamic in the organization of multigene families and other repetitive elements, factors that contribute to karyotypic differentiation and speciation. From a broader perspective, multilocus phylogenies confirm that *Hypancistrus* forms a monophyletic genus within the *Peckoltia* Clade (Lujan et al. 2015), corroborating its evolutionary proximity to other recent lineages of the Hypostominae subfamily. Within the framework of speciation genomics, which considers species boundaries as semipermeable and highlights the role of introgression in recently diverged lineages (Harrison and Larson 2014; Seehausen et al. 2014), the patterns observed here in *Hypancistrus* align with the expectations of incomplete reproductive isolation and gene flow between closely related species. Thus, the comparison with other Loricariidae provides an essential interpretive framework for understanding the patterns detected in the Middle Xingu, a region where the family reaches its greatest diversity and where recent speciation appears to be directly associated with the basin's complex geomorphological history.

The finding that hybrids carry *H. zebra* and *H. seideli* maternal lineages highlights the risks of genetic introgression, particularly for *H. zebra* , an endemic species classified as the IUCN category Critically Endangered (CR) on the Red List of endangered fish species evaluated by the Brazilian government (ICMBio 2018). Hybridization may result in introgression, diluting unique genetic traits and, in extreme cases, contributing to lineage extinction (Mallet 2007; Allendorf et al. 2019). If hybrids are fertile, the potential for genetic dilution of parental species becomes an even greater conservation threat. Such impacts have already been documented in other Neotropical fishes, such as *Prochilodus harttii* (Prochilodontidae), whose genetic integrity was compromised by introgression with non‐native species introduced into its natural basin (Sales et al. 2017). While our study focuses on in situ hybridization, the ability of Loricariidae to hybridize also raises significant applied concerns globally. For instance, the potential impact of invasive loricariids (e.g., *Pterygoplichthys*) hybridizing via aquaculture escapees in non‐native ecosystems represents a critical avenue for future research (Ganguly and Umapathy 2024). Locally, however, the Belo Monte hydropower dam, by altering the hydrological regime of the Volta Grande do Xingu, may be acting as a catalyst for changes in the contact and mating dynamics of *Hypancistrus* species. The magnitude and direction of this influence must be investigated: the hybridization zone may have expanded if the alterations facilitated species encounters; contracted if ecological barriers were created; or remained stable. Testing these hypotheses requires a detailed temporal approach, integrating pre and post‐impact sampling, analyses with biparental markers, and ecological modeling, in order to understand the plasticity and limits of this phenomenon under scenarios of anthropogenic environmental change.

The economic dimension of hybridization in this system is highly relevant, as loricariids hold great commercial value in the global aquarium market. This complexity is further compounded by the fact that, although the capture and commercialization of *H. zebra* are strictly prohibited by law, this restriction has paradoxically made it one of the most valued and heavily trafficked ornamental fishes globally (Sousa et al. 2021). In contrast, the more widespread *H. seideli* and other loricariids are widely and legally traded, creating conditions prone to enforcement confusion. This ambiguity is exacerbated because rare morphological variants, whose collection and export from the Xingu have been documented since the 1980s (Seidel 2008; Camargo et al. 2013), are frequently marketed under “L‐number” codes rather than formal taxonomic names (Stawikowski 1990; Glaser and Glaser 1995). As a result, the overlap between protected and legally permitted specimens, combined with informal nomenclature, severely hinders trade monitoring and can facilitate the illegal export of threatened lineages under the guise of legality (Sousa et al. 2021).

Furthermore, recent surveys reveal a severe in situ depletion of wild stocks, with *H. yudja* populations suffering an estimated decline of over 80% due to habitat degradation (Sousa et al. 2025). In this critical context, the complete mitogenomes established here provide essential molecular tools for in situ conservation. They enable the monitoring of the genetic integrity of remaining wild stocks, the delineation of hybridization zones, and the accurate distinction between pure endemic lineages and hybrids, fundamental steps for enforcing trade regulations and managing the biodiversity of the Xingu River.

The integration of mitochondrial, phylogenetic, and nuclear evidence reinforces the hypothesis that hybridization in the Xingu involves evolutionarily distinct *Hypancistrus* lineages that currently share a secondary contact zone with gene flow. Understanding these dynamics is essential for conservation strategies. Protecting *H. zebra* and *H. seideli*, as well as investigating the role of *H. yudja* as a paternal parent through nuclear markers (such as SNPs or microsatellites), are fundamental steps to assess the real risk of introgression and to support management strategies aimed at preserving the genetic diversity of these endemic species from the Volta Grande do Xingu.

## Author Contributions

**Franciele F. Kerniske:** writing – original draft (equal). **Bruno F. Melo:** writing – original draft (equal). **Leandro M. Sousa:** writing – original draft (equal). **Tiago M. Degrandi:** writing – original draft (equal). **Roberto F. Artoni:** writing – original draft (equal).

## Funding

This work was supported by Coordenação de Aperfeiçoamento de Pessoal de Nível Superior‐Brasil (CAPES), 88887.706125/2022‐00.

## Disclosure

Benefit‐sharing statement: Benefits from this research accrue from the sharing of all data and results on public databases as described above, making them accessible to the broader scientific community. The research also addresses a priority conservation concern for endemic and endangered species in the Xingu River basin.

## Ethics Statement

The research was conducted following the guidelines of the Conselho Nacional de Controle da Experimentação Animal (CONCEA). All procedures were authorized by the Comissão de Ética no Uso de Animais da Universidade Federal do Pará (protocol CEUA No. 6895300622) and the collection of specimens was authorized by SISBIO/MMA license No. 79124‐1.

## Conflicts of Interest

The authors declare no conflicts of interest.

## Data Availability

The mitochondrial genome sequences generated in this study have been deposited in GenBank under accession numbers PX394833–PX394848. All datasets, including sequence alignments, phylogenetic trees, and input files, have been deposited in the Dryad Digital Repository and are available under the DOI: https://doi.org/10.5061/dryad.7pvmcvf6p.
